# Scalar position, dislocation analysis and outcome in CI reimplantation due to device failure

**DOI:** 10.1007/s00405-022-07315-9

**Published:** 2022-02-28

**Authors:** R. Beck, K. Shiraliyev, S. Arndt, A. K. Rauch, A. Aschendorff, F. Hassepass, M. C. Ketterer

**Affiliations:** Faculty of Medicine, Department of Otorhinolaryngology–Head and Neck Surgery, Medical Center, University of Freiburg, Killianstrasse 5, 79106 Freiburg, Germany

**Keywords:** Reimplantation, Cochlear morphology, Technical upgrade, Scalar position, Speech perception

## Abstract

**Objective:**

Due to increasing indication for cochlear implantation (CI), reimplantation and technical upgrades their consequences are a special focus in CI surgery research. The aim of this study is to examine the indication and influences on both morphological position of the electrode array and audiological outcome following reimplantation.

**Design:**

This is a retrospective analysis of adult CI patients reimplanted between 2004 and 2019. We evaluated the scalar position in pre- and postoperative cone beam computed tomography (CBCT) after CI and reimplantation and examined the indication for and the audiological outcome following reimplantation.

**Results:**

The reimplanted patients showed stable and comparable audiological results for monosyllables and numbers for best fitted situation before and following reimplantation. Technical upgrades did not result in a significant improvement of speech perception. CBCT scans of reimplanted ears did not show significant increased rates of scalar dislocation or partial insertion.

**Conclusion:**

Even with a technical upgrade, reimplantation does not improve speech perception outcome in CI patients. Therefore, the indication to reimplant should be approved critically. Reimplantation does not lead to a significantly increased risk for partial insertion, scalar dislocation or diminished electrode array insertion angle.

## Introduction

Due to extended indication and ascertained benefit in cochlear implant (CI) surgery for single-sided deafness [1], asymmetric hearing loss [2, 3] and elderly patients [4], complications and the necessity of reimplantation will increase over the coming years. In addition, more and more patients first implanted in the early 90 s need reimplantation due to a technical dropout with loss of function (i.e. hard failure) of their 20–30-year old implant. Zeitler et al. [5] described hard failure as the most common reason for revision CI surgery, with a range between 40 and 80% of reimplantations. An integrity test of the implant is necessary in cases of supposed implant failure. For the evaluation of technical and audiological defects of the implant, Battmer et al. [6] introduced the “classification of reliability for cochlear implant receiver stimulators” and recommended reimplantation for level B2 (no or reduced clinical benefit of the implanted device) and higher.

Studies examining indication and especially outcome for reimplantation in CI surgery are rare. Nevertheless, this topic needs more focus because of extended CI surgery indications and the growing number of implantations. Due to increasing implantation rates in elderly patients, not only hard device failure, but also fall-related head injuries and device damage will be of increasing interest [7, 8]. Furthermore, the results of speech perception in reimplanted CI patients differ in the existing literature and are highly variable. Other studies described incomplete insertion in 7–18% of the reimplanted patients [9, 10, 11]. There are few studies focusing on the number of incomplete insertions and number of electrodes inserted in reimplant CI surgery [6, 10, 12]. Earlier studies showed that specific position of the electrode array results in a better audiological outcome (Aschendorff et al. [13]), but the studies did not include patients undergoing reimplantation. The aim of this study is to examine reimplant-CI surgery due to device failure with respect to postoperative outcome, scalar position and angular insertion depth of the electrode array.

## Methods

### Study and subjects

We performed a retrospective review of patient charts in the Cochlear Implant Database to identify all patients who underwent CI reimplantation between 2004 and 2019 at our quaternary medical university hospital (Department of Otorhinolaryngology, Head and Neck surgery at the Implant Center of the University Hospital Freiburg). All implant candidates initially suffered from profound bilateral hearing loss without sufficient speech discrimination using hearing aids and were 18 years or older at time of revision surgery. We used the patient’s charts to compare audiological measurements, etiology, side of implantation and more.

We performed postoperative imaging by cone beam computed tomography (CBCT) (DynaCT-equipped Axium Artis dTA angiography unit (Siemens Co., Erlangen, Germany)) with a digital flat-panel detector and measured cochlear morphology, scalar position and insertion angle following CI as described by Ketterer et al. [14, 15]. Imaging was performed following first implantation and following reimplantation. Furthermore, we examined speech perception in a standard clinical setting and used the Freiburg monosyllables and numbers tests before and following first CI surgery as well as before and following reimplantation for best fitted situation. We evaluated open set speech perception in a soundproof chamber using the Freiburg monosyllables and numbers test with presentation at a volume of 65 dB SPL in quiet and we scored speech discrimination in percentage correct. No patient underwent reimplantation on more than one ear.

### Statistics and ethics committee

Statistical analysis was performed using Gnu R statistical computation and graphics system (ANOVA, Tukey’s Honest Significant Difference; GNU R, Version 3.0.3, Core Team, Vienna, Austria, http://www.R-project.org). The level of significance was set at 5.0%.

This retrospective study took place in the department of Otorhinolaryngology, Head and Neck surgery at the Implant Center of the University Hospital Freiburg. The study was approved by the Hospital Ethics Committee (Number: 406/19) according to the declaration of Helsinki (Washington, 2002). We registered this study in the German Clinical Trials Register (www.drks.de/ DRKS number: DRKS00019807).

## Results

35 patients initially implanted between 1993 and 2011 were included. Mean age at first implantation was 36.5 years, mean age at reimplantation was 45.8 years. The interval between activation and reimplantation was 9.3 years (SD 7.2 years). Table 1 shows the distribution of the study cohort. 17 of the 35 included patients received a technical upgrade of the implant and/or the sound processor (see Table 1). All patients underwent reimplantation between 2004 and 2019. We excluded all reimplanted patients who were reimplanted due to medical reasons (e.g. infection, inflammation, cholesteatoma) to create a clean study cohort with the focus on reimplanted patients with device failure only.

**Table 1:** Study cohort and distribution table of the included reimplanted CI patients (*n* = 35) (*SP*  sound processor; *CI*  cochlear implant). (*x*  no comparison of scalar position and insertion depth available)

Patient	Side	First implant	First SP	Device failure	Second implant	Second SP	Upgrade	Partial insertion
1	Left	CI22M	Spectra	Unknown	CI512	Freedom SP	Yes	No
2	Left	CI22M	Spectra	Unknown	CI24RECA	Freedom SP	Yes	No
3	Left	CI22M	Spectra	Unknown	CI24RECA	Freedom SP	Yes	Yes
4	Right	CI22M	Freedom SP	Cracked electronic assembly	CI522	CP910	Yes	X
5	Right	Hybrid L	CP910 Hybrid	Unknown	CI522	CP1000	Yes	No
6	Left	CI512	Freedom SP	Unknown	CI24RECA	CP810	No	No
7	Left	CI22M	Freedom SP	Unknown	CI622	CP1000	Yes	X
8	Right	CI512	CP910	Electronic failure, hermetic failure	CI512	CP950/Kanso	No	No
9	Right	CI24RCA	CP910	Electrode anomaly	CI522	CP910	Yes	No
10	Left	CI22M	Freedom SP	Unknown	CI522	CP910	Yes	No
11	Right	CI24RST	Esprit 3G	Open circuit electrode	CI522	CP910	Yes	X
12	Right	CI22M	Freedom SP	Unknown	CI422	CP910	Yes	X
13	Left	C40 + Medel	Tempo	Unknown	Pulsar	Opus 2	Yes	X
14	Left	Unbekannt	Esprit 22	Unknown	CI24REST	Freedom SP	X	No
15	Left	CI24M	Freedom SP	Electode anomaly, insulation failure	CI522	CP910	Yes	X
16	Right	CI512	CP810	Hermetic failure	CI24RECA	CP810	No	X
17	Left	CI24M	Esprit 3G	Unknown	CI24REST	Freedom SP	Yes	No
18	Left	CI24RCS	Freedom SP	Unknown	CI24RECA	Freedom SP	No	No
19	Right	CI512	CP810	Unknown	CI512	CP1000	No	No
20	Left	CI24RE CA	Freedom SP	Electrode array malfunc-tion	CI512	CP810	Yes	X
21	Right	CI24RE CA	Freedom SP	Breach in the electrical insulation	CI24RECA	Freedom SP	No	No
22	Left	Sonatati	Opus 2	Unknown	Flex Soft	Opus 2	yes	no
23	Right	HiRes90K/HiFokus	Harmony	Unknown	HiRes Ultra 3D MS	Naida CI Q90	No	No
24	Left	CI512	CP810	Hermetic failure	CI24RECA	CP810	No	No
25	Left	Sonatati100	Opus 2	Unknown	Synchrony	Sonnet EAS	Yes	No
26	Left	CI512	CP810	Unknown	CI512	CP910	No	No
27	Left	CI512	CI810	Unknown	CI512	CP1000	No	No
28	Left	CI512	CP810	Electronic failure, hermetic failure	CI512	CP1000	No	No
29	Left	CI512	CP810	Electronic failure, hermetic failure	CI24RECA	CP810	No	No
30	Left	CI512	CP810	Hermetic failure	CI512	CP950/Kanso	No	No
31	Left	CI512	CP810	Hermetic failure	CI24RECA	CP810	No	No
32	Left	CI512	CP810	Unknown	CI24RECA	CP810	No	No
33	Left	CI512	CP810	Hermetic failure	CI512	CP910	No	No
34	Right	Flex 28	Opus 2	Unknown	CI512	CP910	No	X
35	Left	C40 + Medel	Sonnet EAS	Unknown	Synchrony	Sonnet EAS	Yes	X

**Fig. 1:**
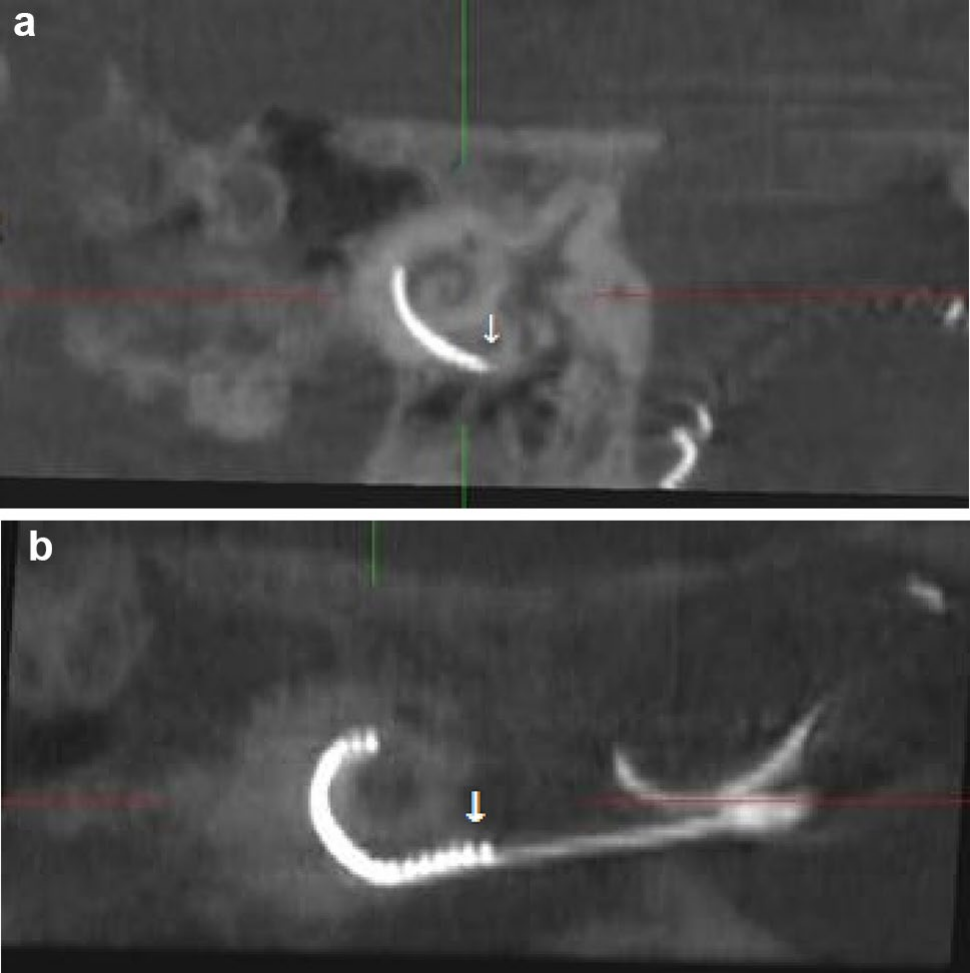
**a** Partial insertion at initial implantation with decreased cochlear coverage of 180° (electrode array: CI22 + 10 Cochlear™) with 15 of 22 active electrodes within the cochlea (arrow = extracochlear electrodes). **b** Partial insertion at reimplantation with decreased but improved cochlear coverage of 240° (electrode array: CI522 Cochlear™) with 20 intracochlear electrodes of 22 active electrodes (arrow = extracochlear electrodes)

Table 2 describes the scalar position and angular insertion depth of the electrode array evaluated in CBCT scans. CBCT scans for 24 patients were pre- and postoperatively available and were analyzed. Imaging via CBCT was established in 2004 in our department as described by Aschendorff et al. [16]. Therefore, earlier implanted or reimplanted patients underwent postoperative X-ray imaging control and were not analyzed further. One patient (4.2%) showed a partial insertion due to partial obliteration before and following reimplantation with an initial angular insertion depth of 180° (Fig. 1a; electrode array CI 22 + 10 of Cochlear™). Figure 1b shows the post-reimplant CBCT with an increased insertion angle of 240° (CI 522; Cochlear™) compared to initial implantation.

**Table 2:** Scalar position of the electrode array evaluated by postoperative CBCT and rate of partial insertion at reimplantation:

Scalar position in CBCT following first CI	ST: 58.3%	SV: 8.3%	TD: 33.3%
Scalar position following reimplantation (0.6%: scans not evaluable)	ST: 60.2%	SV: 8.8%	TD: 30.4%
Insertion angle 1	Mean: 349.4°	Min: 156.3°	Max: 495.8°
Insertion angle 2	Mean: 337.5°	Min: 209.8°	Max: 520.5°

The data of Table 2 demonstrate comparable and not significantly different rates of scalar position (ST versus SV versus TD). Therefore, we hypothesize that due to a fibrosis following initial insertion, scalar position stays the same in reimplantation. Furthermore, angular insertion depth is not diminished comparing initial implantation and reimplantation (Table 2).

**Fig. 2:**
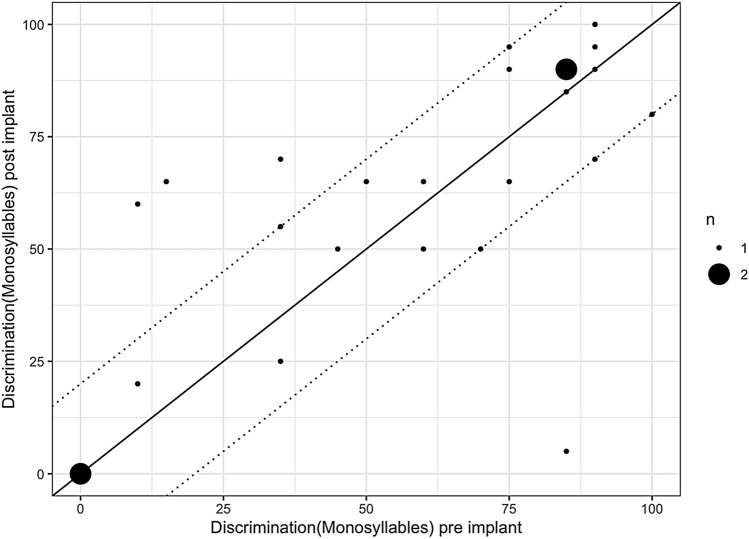
Comparing the best results for monosyllables following CI to following reimplantation patients did not show diminished results (*p* = 0.534) (the area between the two dotted lines gives the test–retest reliability (Winkler and Holube 2016)) (legend shows *n* = 1 patient or *n* = 2 patients)

**Fig. 3:**
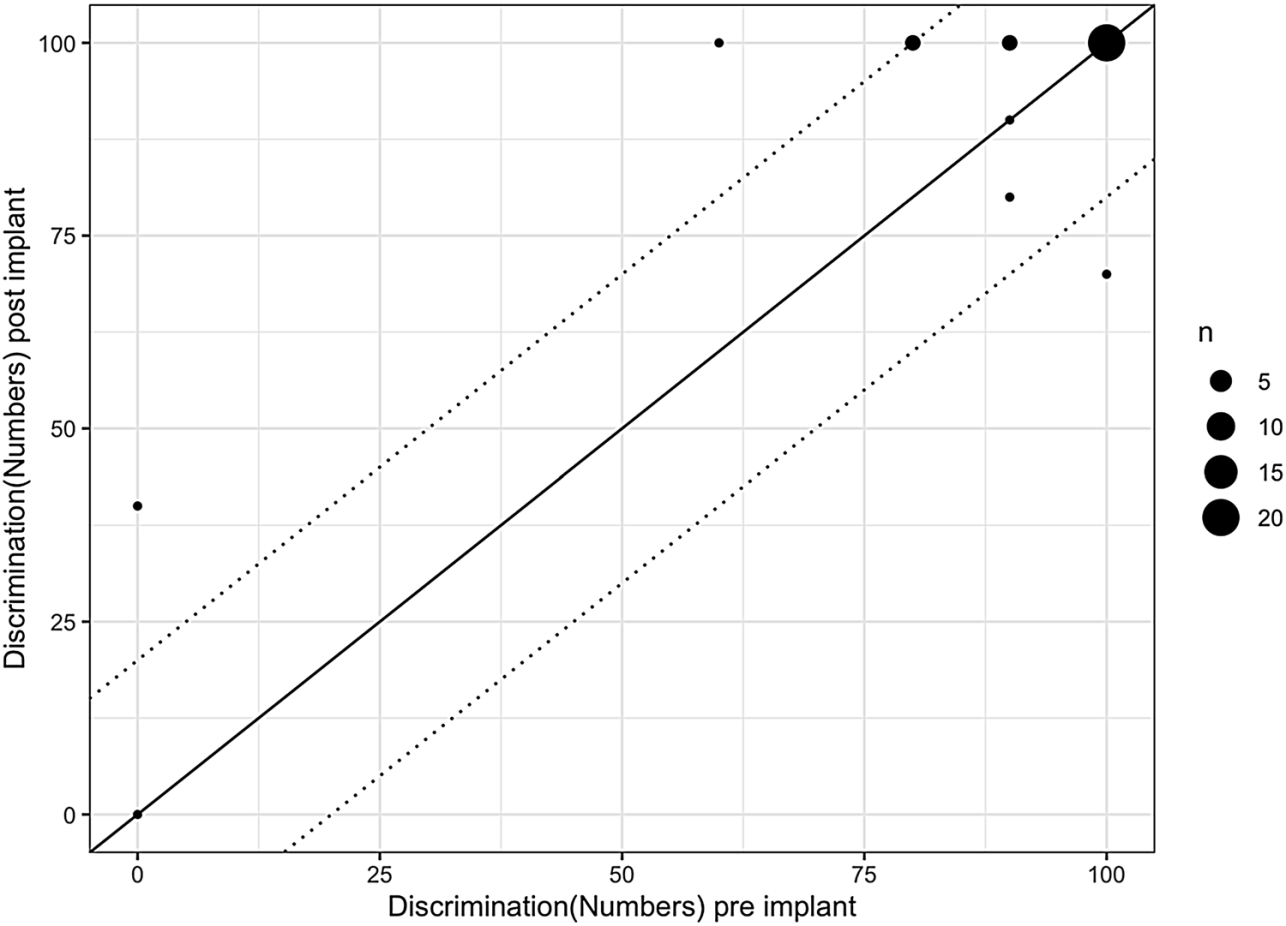
Patients show stable results for numbers following reimplantation compared to their best result following first implantation (*p* = 0.169) (the area between the two dotted lines gives the test–retest reliability (Winkler and Holube 17)) (legend shows *n* = 5–20 patients)

Figure 2 shows the best fitted result for monosyllables following reimplantation compared to best fitted results following first implantation (the area between the two dotted lines gives the test–retest reliability described by Winkler and Holube [17]. The patients neither significantly improved nor showed significantly diminished results (*p* = 0.534). One patient showed significantly worse speech perception outcomes. This patient did not undergo the usual rehabilitation program and best fitted post-reimplant monosyllables were not available. Furthermore, the reimplanted patients included in this study show stable results for numbers (Freiburg number test) compared to their best fitted examination following first implantation (Fig. 3) (*p* = 0.169). Calculating speech perception results for patients who underwent a technical upgrade separately, we could detect a significant improvement neither for numbers (*p* = 0.806) nor for monosyllables (*p* = 0.0796).

## Discussion

### Speech perception following reimplantation

All reimplanted patients included in this study underwent reimplantation due to device failure. 17 patients received a technical upgrade (see Table 1). Comparing speech perception results in monosyllables and numbers, we could not find significantly changed results following reimplantation. Even those patients who received a technical upgrade did not show better results following reimplantation. It has to be mentioned that the reason for not significantly different results for numbers might also be a ceiling effect. However, we could not find a significant decrease in post-reimplant speech perception results in monosyllables or numbers.

Previous studies showed that technical upgrades with new sound processors can lead to more than 10% better speech perception results with the same implant. Rauch et al. [18] reported that patients with new sound processors showed a significant improvement in speech perception independent of age. Therefore, even with new sound processors and new implant, reimplantation solely for technical upgrade should be carefully indicated.

Nevertheless, speech perception outcome following reimplantation in previous literature differs. Manrique-Huarte et al. [19] described 64% improved and 9% stable disyllabic word recognition scores compared to before CI reimplantation. Reis et al. [7] examined whether the audiological outcome after implantation interacts with the speech perception score following reimplantation but could not find a significant influence. Unsuccessful outcome was defined as patients whose speech perception score did not improve from their last measured score to reimplantation and the authors reported 44 successful and 9 unsuccessful cases. Rivas et al. [20] described 34 reimplanted patients and 65% with better, 32% with the same and 3% with worse speech results following reimplantation. Mahtani et al. [21] reported on 25 reimplanted patients, but only tested 16 patients in noise. They reported 8% poorer and 36% better results following reimplantation. Battmer et al. [6] reported 30% worse speech discrimination scores compared to before reimplantation. Nevertheless, most studies [7, 19, 20 and 21] report better speech perception results in the majority of the reimplanted patients. These studies included all reimplanted patients without cohorting them by indication. In contrast, we only included patients with device failure and excluded patients reimplanted following trauma or infection. Further studies are needed with clear groups of indication and larger study cohorts. Nevertheless, reimplantation should be discussed critically with the patient and information about possible speech perception loss is necessary. In light of our data, technical upgrade without a device failure or an infection is only indicated in the rare case of missing technical support of very old implants. The upgrade of sound processors in CI patients may contribute to an improved speech understanding [18]. Following reimplantation, we did not see a similar effect but rather a comparable performance. Reasons for this missing improvement may be an additional intracochlear trauma due to the explantation and reinsertion of an electrode array that induces a loss of neural elements. In addition, newer sound processors are often characterized by an increased stimulation rate but this may not be well tolerated by an auditory system used to lower stimulation rates. Both hypotheses may contribute to our observations in various and individual extent.

### Scalar position and insertion angle following reimplantation

This study demonstrates that scalar position rates are comparable before and following reimplantation. The risk for partial insertion, scalar dislocation and diminished angular insertion depth is not higher at reimplantation. The surgeons always intended to reach an identical electrode position and angular insertion depth to maintain a comparable hearing sensation with regard to pitch. Authors reported of neo-ossification, extracochlear factors like adhesions or fibrotic bands within the mastoid or trauma [19, 23]. Manrique-Huarte [19] speculated that reimplantation is safe when depth of insertion is equal or higher, so that speech perception is better or the same. They recommended minimally traumatic electrode arrays and surgical techniques. Reis et al. [7] described that all unsuccessfully reimplanted patients had complications during initial CI surgery or an incompletely inserted electrode array. Sterkers et al. [24] described reimplantation in 45 children and performed CBCT following reimplantation. They inserted 6 different electrode arrays of 3 manufacturers and reported 42 ST insertions, 1 SV insertion and 2 partial insertions. Furthermore, they described no significant difference between insertion angle before and after reimplantation. We can confirm these findings in adults and could not find a significant diminished angular insertion depth and comparable scalar positions for reimplanted electrode arrays, probably due to intracochlear fibrosis.

## Conclusion

We only recommend reimplantation in case of device failure or infection as we did not see significant improvement of speech perception, even with a technical upgrade.

Reimplantation does not lead to a significantly increased risk for partial insertion, scalar dislocation or diminished cochlear coverage.
